# Self-supervised disturbing feature reconstruction network for mangrove biomass estimation with limited data

**DOI:** 10.3389/fpls.2025.1623458

**Published:** 2025-09-03

**Authors:** Jun Hao, Xiaowei Xu, Haiyan Xu, Gang Xu

**Affiliations:** ^1^ School of Environment and Spatial Informatics, China University of Mining and Technology, Xuzhou, China; ^2^ College of New Energy Equipment, Zhejiang College of Security Technology, Wenzhou, China; ^3^ Wenzhou Future City Research Institute, Wenzhou, China; ^4^ Wenzhou Forestry Technology Extension and Wildlife Protection Management Station, Wenzhou, China; ^5^ Wenzhou Key Laboratory of Natural Disaster Remote Sensing Monitoring and Early Warning, Wenzhou, China; ^6^ Wenzhou Collaborative Innovation Center for Space-borne, Airborne and Ground Monitoring Situational Awareness Technology, Wenzhou, China

**Keywords:** mangrove biomass estimation, self-supervised learning, disturbed feature reconstruction, multi-view convolution neural network, deep learning

## Abstract

Accurate estimation of mangrove biomass is significant for ensuring the mangrove ecosystem’s productivity and global carbon cycling. Although well-known deep neural networks (DNNs) have been successfully applied in mangrove biomass estimation using remote sensing data, the key problem of data scarcity is not addressed very well for existing methods. Thus, a novel DNN called self-supervised disturbing feature reconstruction network (SSDFRN) is constructed in this article for mangrove biomass estimation with limited data. First, a disturbing feature reconstruction-based self-supervised learning (DFRSSL) method based on random feature shuffle and disturbing feature reconstruction is proposed for solving the data scarcity problem. In addition, a multi-view convolutional neural network (MVCNN) is constructed by stacking several multi-view cascaded convolution modules (MVCCMs), which effectively enhances feature learning performance and improves mangrove biomass estimation accuracy. The mangrove biomass dataset obtained from Ximen Island (28° 21′ N, 121° 10′ E) is used in this study to verify the outperformance of SSDFRN. The experimental results illustrate that SSDFRN is effective in deep feature learning and mangrove biomass estimation with limited data.

## 1 Introduction

Mangrove plays a significant role in maintaining biodiversity, carbon sequestration, and carbon storage (Tran et al., 2022). Accurate estimation of aboveground biomass (AGB) is an important part of the mangrove ecosystem carbon cycle, which is conducive for assessing the carbon sink potential of the mangrove ecosystem (Sliva et al., 2024). The traditional mangrove survey method is destructive, costly, and inefficient, which greatly restricts the efficiency of estimating and monitoring mangrove biomass distribution (Morais et al., 2021; Zhang et al., 2022). Due to the interference of various external environmental factors (e.g., growing environment, climatic factors, and geographical position), it is difficult to promptly and accurately estimate mangrove biomass using the traditional survey method.

Remote sensing technology has the advantages of a large spatial scale, strong timeliness, and high efficiency (Zhao et al., 2023; Hazmy et al., 2024), which greatly saves the manpower and material resources required by traditional investigation (Gao et al., 2022) (Muhd-Ekhzarizal et al., 2018). employed simple and multilinear regression methods for the estimation of AGB in the entire study area using remote sensing images (Pandey et al., 2019). utilized logarithmic and polynomial (second degree) models for mangrove biomass estimation, and the study shows that normalized difference vegetation index (NDVI) and enhanced vegetation index (EVI) derived from satellite images are effective indexes for biomass estimation. However, traditional remote sensing-based linear regression and non-linear regression models have poor performance in estimating mangrove biomass and are not suitable for practical application.

Machine learning (ML) [e.g., support vector machine (SVM) (Li et al., 2023), random forest (RF) (Xiao et al., 2024), and support vector regression (SVR) (Rahimikhoob et al., 2023)] learns the relationship between input and output by fitting a flexible model (Teshome et al., 2023), which has been widely used in mangrove biomass estimation (Tian et al., 2021; Hao et al., 2024) (Selvaraj and Perez, 2023). developed an RF-based spatial estimation approach to assess mangrove AGB using the Google Earth Engine (GEE) platform (Bui et al., 2024). proposed an ML-based (i.e., LightGBM and XGBoost) AGB estimation method, and the hyperparameters were tuned by Bayesian-based optimizers and a novel Tasmanian Devil optimization algorithm (Tian et al., 2022). analyzed the quantitative relationship between invasive mangrove biomass and hydrological units using different ML algorithms (Do et al., 2022). proposed a principal component analysis-based ML technique for estimating the mangrove AGB (Rijal et al., 2023). developed a novel ML-based mangrove aboveground carbon (AGC) estimation technique based on extreme gradient boosting and genetic algorithm analyses (Hu et al., 2024). proposed an SVM-based AGB estimation method using remote sensing data (Luo et al., 2024). developed a novel AGB estimation method using SVR, and the parameters of SVR were optimized by the global best particle swarm algorithm. However, the feature learning ability of traditional ML methods is limited due to their shallow network structures. In addition, RF-based methods typically cannot make accurate estimations when the training samples are limited, and the performance of SVM and SVR models depends heavily on the choice of kernel function (He et al., 2024). Thus, effective feature learning techniques are needed for mangrove biomass estimation based on remote sensing data.

Recently, deep learning (DL) techniques have been widely used in various domains due to their outstanding feature learning capacity (Li et al., 2024; Miao and Yu, 2024). Typical deep neural networks (DNNs) [e.g., deep belief network (DBN), convolutional neural network (CNN), and long short-term memory (LSTM) network] have been successfully applied in mangrove biomass estimation (Akkem et al., 2023) (Chen et al., 2022). proposed a generative adversarial network for data augmentation using Sentinel-2 images and a DBN for deep feature learning and obtaining the salt marsh distribution (Nakajima et al., 2023). proved that the CNN-based estimation technique is outstanding and can be applied for monitoring crop AGB in diverse cultivars (Tian et al., 2024). proposed a novel AGB estimation method based on CNN and LSTM using different remote sensing image data (Talebiesfandarani and Shamsoddini, 2022). developed a novel global-scale biomass estimation method by learning features using CNN, and RF and SVR are used for feature selection (Song and Wang, 2023). proposed a recurrent neural network (RNN)-based method for forest energy estimation (Zhang et al., 2024). proposed a novel framework for AGB estimation using Sentinel-1 synthetic aperture radar (SAR) and Sentinel-2 optical data, where the bidirectional long short-term memory (BiLSTM) neural network is implemented for deep feature learning (Liu et al., 2024). proposed a residual neural network (ResNet)-based model to extract phenological information from wheat and implemented the AGB estimation. Nevertheless, these methods always assume that training samples are sufficient and rely heavily on the quantity and quality of data. When the data are limited, these models are prone to overfitting. In the actual scenario, the data scarcity problem is inescapable due to the difficulty of obtaining high-quality mangrove biomass data, which greatly limits the application of these methods.

In order to address the above problems, a novel DNN, called self-supervised disturbing feature reconstruction network (SSDFRN), is proposed for mangrove biomass estimation with limited data in this study. The main contributions of this study are summarized as follows: 1) a self-supervised disturbing feature reconstruction network is proposed for deep feature learning, 2) a disturbing feature reconstruction-based self-supervised learning (DFRSSL) method based on random feature shuffle and disturbing feature reconstruction is developed for solving the data scarcity problem, and 3) a multi-view convolutional neural network (MVCNN) is constructed by stacking several multi-view cascaded convolution modules (MVCCMs), which effectively enhances the feature learning performance and improves the mangrove biomass estimation accuracy. The experimental results on the mangrove biomass dataset obtained from Ximen Island (28° 21′ N, 121° 10′ E) demonstrate the outperformance of SSDFRN for mangrove biomass estimation with limited data.

The remainder of this article is organized as follows. The details about SSDFRN are given in Section 2. The experimental analysis of SSDFRN-based mangrove biomass estimation is implemented in Section 3. Finally, the conclusions are given in Section 4.

## 2 Self-supervised disturbing feature reconstruction network

In this study, SSDFRN is proposed for mangrove biomass estimation with limited data. In particular, first, Landsat 8 remote sensing data and Digital Elevation Model (DEM) data are used for extracting 22 features (i.e., band information, vegetation indexes, texture features, and elevation features). Then, the shuffle window is masked on partial input features randomly for generating auxiliary data and residual data. MVCNN is constructed for deep feature learning from residual data, and learned features are combined with auxiliary features for disturbing feature reconstruction. In the process of DFRSSL, the feature representation ability of the deep network will be greatly enhanced with limited data.

### 2.1 Network structure

**Figure 1:**
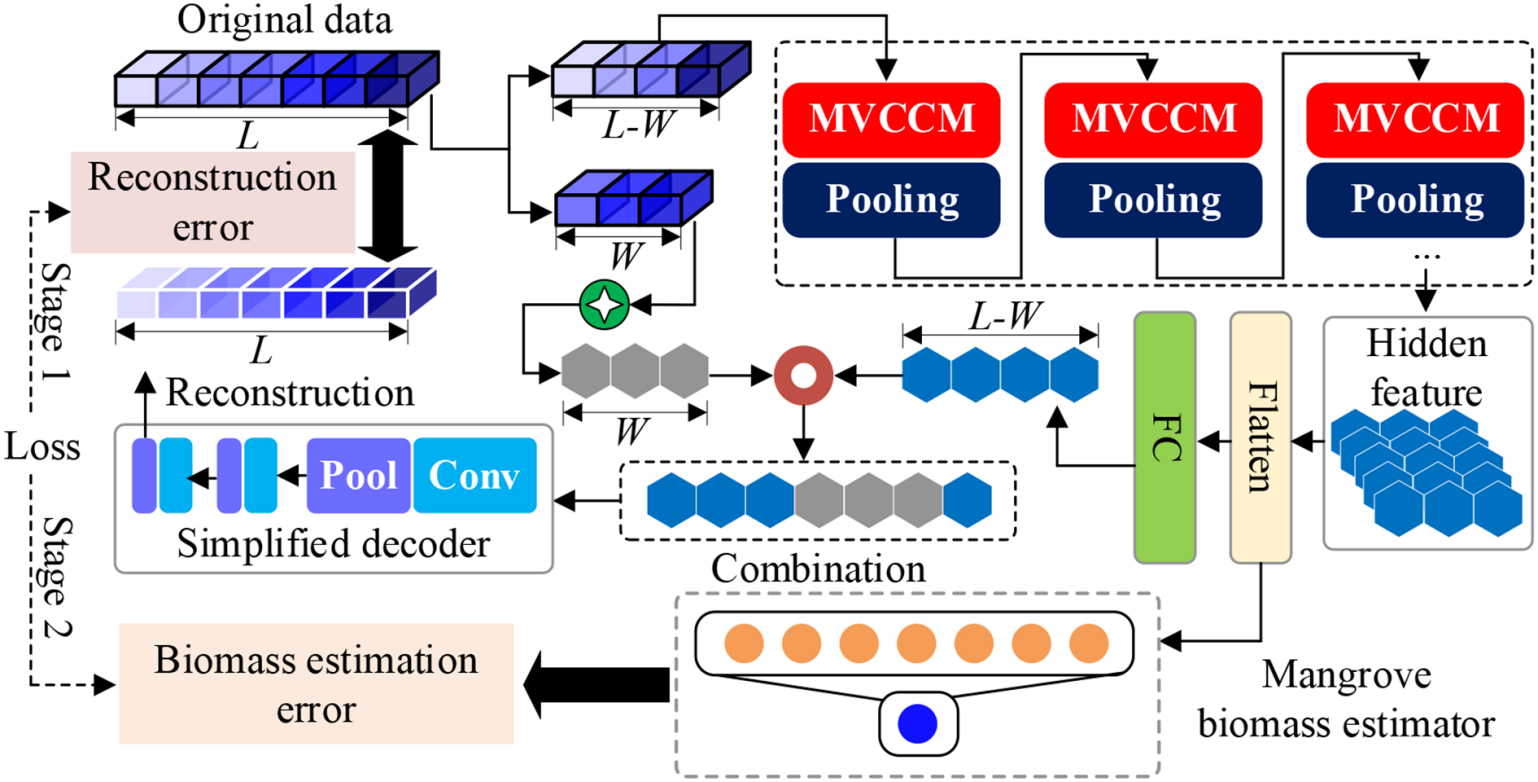
Network structure of SSDFRN. SSDFRN, self-supervised disturbing feature reconstruction network.

The SSDFRN-based mangrove biomass estimation method is shown in **Figure 1**, which includes two stages: DFRSSL and fine-tuning. In **Figure 1**, L denotes the total number of input features, and *W* is the length of the shuffle window. In the stage of DFRSSL, first, limited samples are used to generate plenty of auxiliary data and residual data. Then, MVCNN is implemented for deep feature learning from sufficient residual data. Finally, disturbing feature reconstruction is implemented based on deep features and auxiliary data for solving the problem of feature learning with limited data. In the stage of fine-tuning, learned representations are fed into the biomass estimator for mangrove biomass estimation.

### 2.2 Disturbing feature reconstruction-based self-supervised learning

In the actual scenario of mangrove biomass estimation, the problem of data scarcity is inevitable due to the difficulty of data collection. Traditional mangrove biomass estimation methods highly depend on data quantity and quality, which could limit their applications in a real environment. Thus, DFRSSL is developed in this study for solving the problem of feature learning and mangrove biomass estimation with limited data.

#### 2.2.1 Generation of auxiliary data and residual data

**Figure 2:**
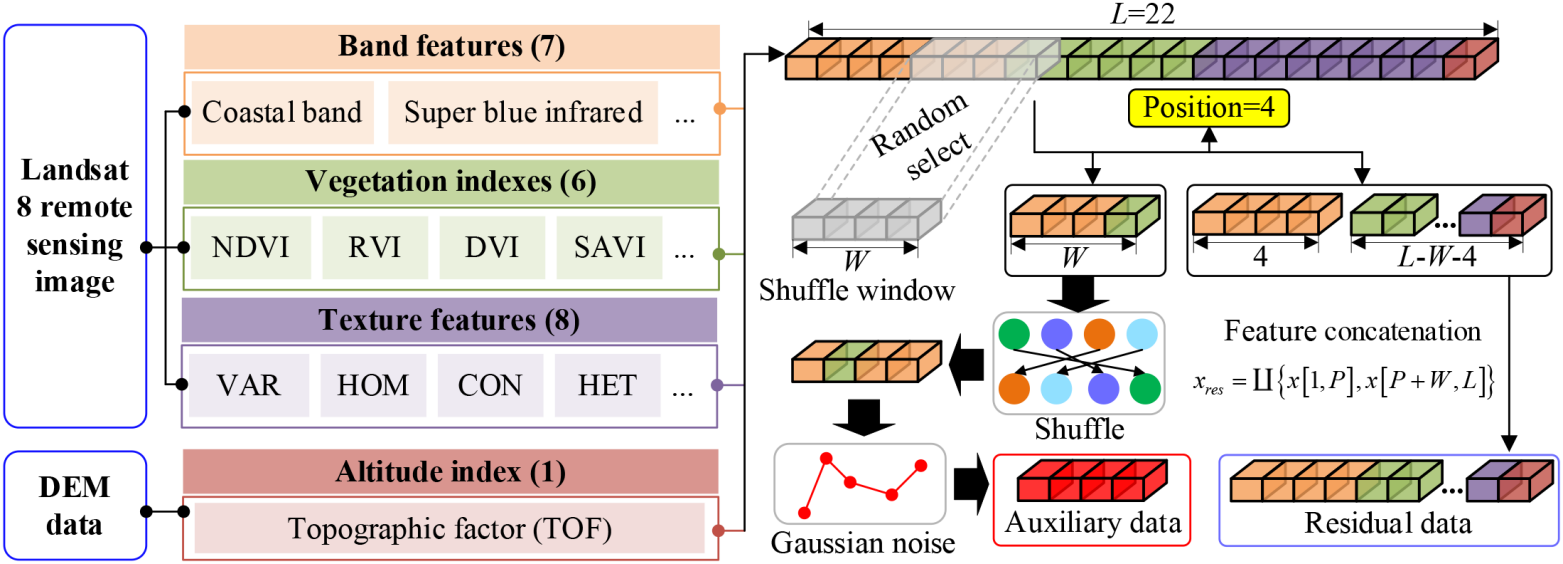
Generation process of auxiliary data and residual data.

In the stage of DFRSSL, first, Landsat 8 remote sensing image and DEM elevation data are used for extracting 22 features (i.e., band information, vegetation indexes, texture features, and elevation features). Then, the shuffle window is masked on partial input features randomly for generating auxiliary data and residual data. This operation has two main functions: 1) the masking method can generate data pairs that are not limited by the scarcity of original data so as to solve the problem of limited data availability. 2) This method largely reduces redundancy and creates a challenging SSL task that requires a holistic understanding of the relationship between all input features (i.e., band information, vegetation indexes, texture features, and elevation features). The generation process of auxiliary data and residual data is shown in **Figure 2**. In particular, a shuffle window with length *W* is used to randomly select *W* features for feature disturbance. In the meantime, Gaussian noise is used to mask the *W* features after the disturbance to simulate the interference of the external environment.

The generation process of auxiliary data is represented as follows:


(1) P=ψ(1∼(L−W−1))

(2) $$\tilde{x}=\dot{N}+\chi (x[P,P+W])$$

where *P* is the randomly selected feature position, ψ(a∼b) indicates that an integer is randomly selected from *a* to *b*, *L* is the total number of input features (*L* = 22 in this study), *W* is the length of shuffle window, $\tilde{x}$ denotes the generated auxiliary data, $\dot{N}$ represents the Gaussian noise, *x*[*a*, *b*] represents features from segments *a* to *b* of the original data, and χ() is the feature disturbance operation. For the unselected feature segments, the residual data are obtained by data concatenation, which is expressed as follows:


(3) $$x_{res}=∐\{x[1,P],x[P+W,L]\}$$

where *x_res_* indicates the residual data, and ∐{ } is the data concatenation operation. It should be noted that the number of auxiliary data and that of residual data are not limited by the size of the original samples, and the original limited data will be greatly enhanced by generating multiple auxiliary and residual data.

#### 2.2.2 Disturbing feature reconstruction-based self-supervised learning

**Figure 3:**
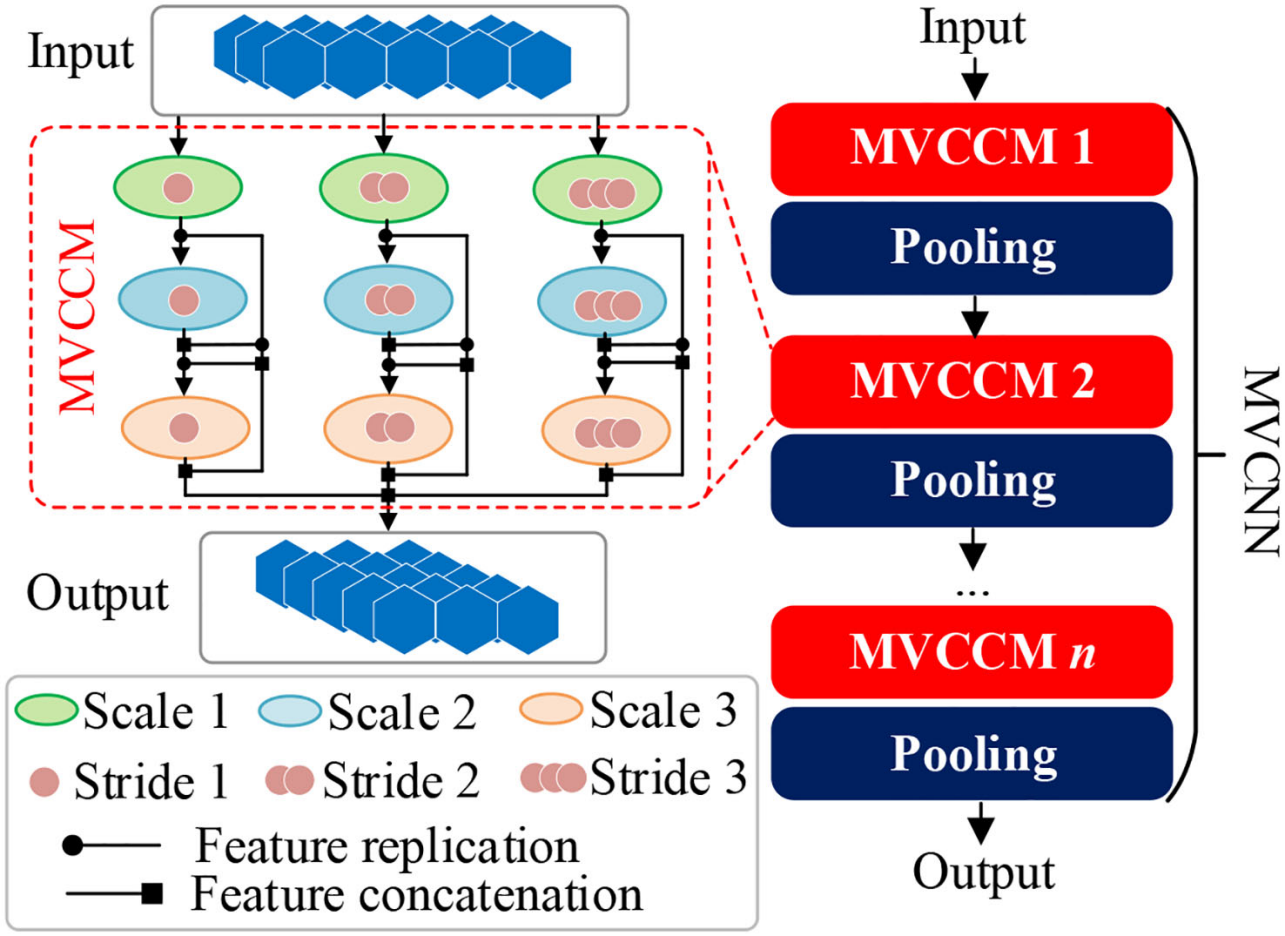
Structure of MVCCM and MVCNN. MVCCM, multi-view cascaded convolution module; MVCNN, multi-view convolutional neural network.

In this study, MVCNN is constructed for deep feature learning from residual data. The structure of MVCNN is shown in **Figure 3**, which is composed of multiple MVCCMs. In **Figure 3**, the numbers of circles in MVCCM signify different sliding strides.

Taking the first MVCCM as an example, the small-scale convolution is first used to obtain small-field features from residual data *x_res_* using different convolution steps, which are calculated as follows:


(4) $$O_{i}^{S}=Conv_{i}^{S}(x_{res})i=123$$

where $O_{i}^{S}$ denotes the *i*th small-view feature and $Conv_{i}^{S}()$ is the small-scale convolution with step size *i*. After obtaining the small-view features, medium-scale convolution is used to obtain medium-view features with different convolution steps, as follows:


(5) $$O_{i}^{M}=Conv_{i}^{M}(O_{i}^{S}),i=1,2,3$$

where $O_{i}^{M}$ denotes the *i*th medium-view feature, and $Conv_{i}^{M}()$ represents the medium-scale convolution with step size *i*. After obtaining the small-view features and medium-view features, large-scale convolution is used to obtain the big-view features based on the cascading convolution, which is calculated as follows:


(6) $$O_{i}^{B}=Conv_{i}^{B}(\Theta \{O_{i}^{S},O_{i}^{M}\}),i=1,2,3$$

where $O_{i}^{B}$ is the *i*th big-view feature, $Conv_{i}^{B}()$ denotes the large-scale convolution with step size *i*, and Θ{ } represents the feature concatenation operation. Finally, the output feature of the first MVCCM is obtained as follows:


(7) $$O^{MVCCM}=\Theta \{O_{1}^{B},O_{2}^{B},O_{3}^{B}\}$$

where *O^MVCCM^* represents the output feature of the first MVCCM. MVCNN is constructed by cascading multiple MVCCMs and pooling layers, and the output feature of MVCNN is represented as follows:


(8) $$O^{MVCNN}=\Phi^{n}\langle Pool(MVCCM)\rangle$$

where *O^MVCNN^* denotes the learned hidden representation, $\Phi^{n}\langle \;\rangle$ indicates the cascading operation of *n* MVCCMs, and *Pool* and *MVCCM* represent the pooling layer and MVCCM, respectively.

After obtaining hidden representations *O^MVCNN^*, the simplified decoder is implemented for disturbing feature reconstruction (He et al., 2022) based on auxiliary data $\tilde{x}$ and corresponding feature position *P*. First, the hidden representation *O^MVCNN^* learned by MVCNN is flattened and then spliced with auxiliary data as the input of simplified decoder. It should be noted that the feature disturbing position is considered in the splicing process. The input of the simplified decoder is obtained as follows:


(9) $$x_{de}=∐\{Flat(O^{MVCNN})[1,P],\tilde{x},Flat(O^{MVCNN})[P+W,L]\}$$

where *x_de_* denotes the input feature of the simplified decoder and *Flat* () indicates the feature flatten operation. In this study, multiple one-dimensional convolution layers and pooling layers are cascaded to construct the simplified decoder, which is represented as follows:


(10) $$O_{de}=\Phi^{n}\langle Pool(Conv(x_{de}))\rangle$$

where *O_de_* represents output feature of the simplified decoder. Finally, the reconstructed feature is obtained as follows:


(11) $$\hat{x}=FC(Flat(O_{de}))$$

where $\hat{x}$ is the output reconstructed feature and *FC* indicates the fully connected layer.

### 2.3 Mangrove biomass estimation

In the stage of fine-tuning, the biomass estimator is constructed based on two fully connected layers for mangrove biomass estimation, as follows:


(12) $$\hat{y}=FC_{2}(FC_{1}(Flat(O^{MVCNN})))$$

where $\hat{y}$ denotes the estimated mangrove biomass and *FC_i_* indicates the *i*th fully connection layer.

### 2.4 Loss function of SSDFRN

In this study, the training process of SSDFRN consists of two stages: DFRSSL and fine-tuning. In the stage of DFRSSL, the loss of SSDFRN is calculated as follows:


(13) $$L^{s1}=\frac{1}{M\times L}\sum_{j=1}^{M}\sum_{i=1}^{L}{\left({\hat{x}}_{j}(i)-x_{j}(i)\right)}^{2}$$

where *L^s^* ^1^ is the loss of SSDFRN in the DFRSSL stage; ${\hat{x}}_{j}(i)$ and $x_{j}(i)$ denote the reconstructed value and actual value of the *i*th feature for the *j*th mangrove sample, respectively; and *M* is the total number of samples.

In the stage of fine-tuning, the loss of SSDFRN is calculated as follows:


(14) $$L^{s2}=\frac{1}{M}\sum_{j=1}^{M}{\left({\hat{y}}_{j}-y_{j}\right)}^{2}$$

where *L^s^* ^2^ is the loss of SSDFRN in the fine-tuning stage, and ${\hat{y}}_{j}$ and $y_{j}$ represent the estimated mangrove biomass and the actual mangrove biomass of the *j*th sample, respectively. The training process of SSDFRN is shown in **Table 1**.

**Table 1:** Training process of SSDFRN.

Training of SSDFRN
**Input:** *x*: 22 features extracted from Landsat 8 remote sensing image and DEM elevation data;
*y*: actual mangrove biomass value.
Init parameters of SSDFRN.
**Stage 1: DFRSSL**
*** For each training epoch*:**
Randomly select *W* features from *x* and record the position *P* by Equation 1;
Generate auxiliary data $\tilde{x}$ by Equation 2;
Combine remaining features as the residual data *x_res_* by Equation 3;
Obtain the hidden representation *O^MVCNN^* from *x_res_* by Equations 4–8;
Combine auxiliary data $\tilde{x}$ and hidden representation *O^MVCNN^* by Equation 9;
Obtain reconstructed feature $\hat{x}$ by Equations 10, 11.
Compute loss of SSDFRN in the stage of DFRSSL by Equation 13;
Update parameters of SSDFRN.
*** End***
**Stage 2: Fine-tuning**
*** For each training epoch:***
Obtain deep features by MVCNN optimized in stage 1;
Obtain estimated mangrove biomass by Equation 12;
Compute mangrove biomass estimation error by Equation 14;
Update parameters of mangrove biomass estimator.
*** End***

SSDFRN, self-supervised disturbing feature reconstruction network; DFRSSL, disturbing feature reconstruction-based self-supervised learning; MVCNN, multi-view convolutional neural network.

## 3 Experimental analysis

In this section, the mangrove biomass dataset obtained from Ximen Island (28° 21′ N, 121° 10′ E) is used for verifying the effectiveness of SSDFRN in mangrove biomass estimation. The research area is located north of Wenzhou City, which experiences a subtropical oceanic monsoon climate. The study area is mild and humid throughout the year, with abundant rainfall. The experiment hardware environment is as follows: CPU, Intel^®^ i7-10875H; GPU, RTX2060 6G. The software environment is as follows: programming language, Python3.7; compiler, Pycharm2022.3.2; framework, Tensorflow-gpu2.6.0+Cuda10.0.

### 3.1 Experimental description

#### 3.1.1 Data description

**Figure 4:**
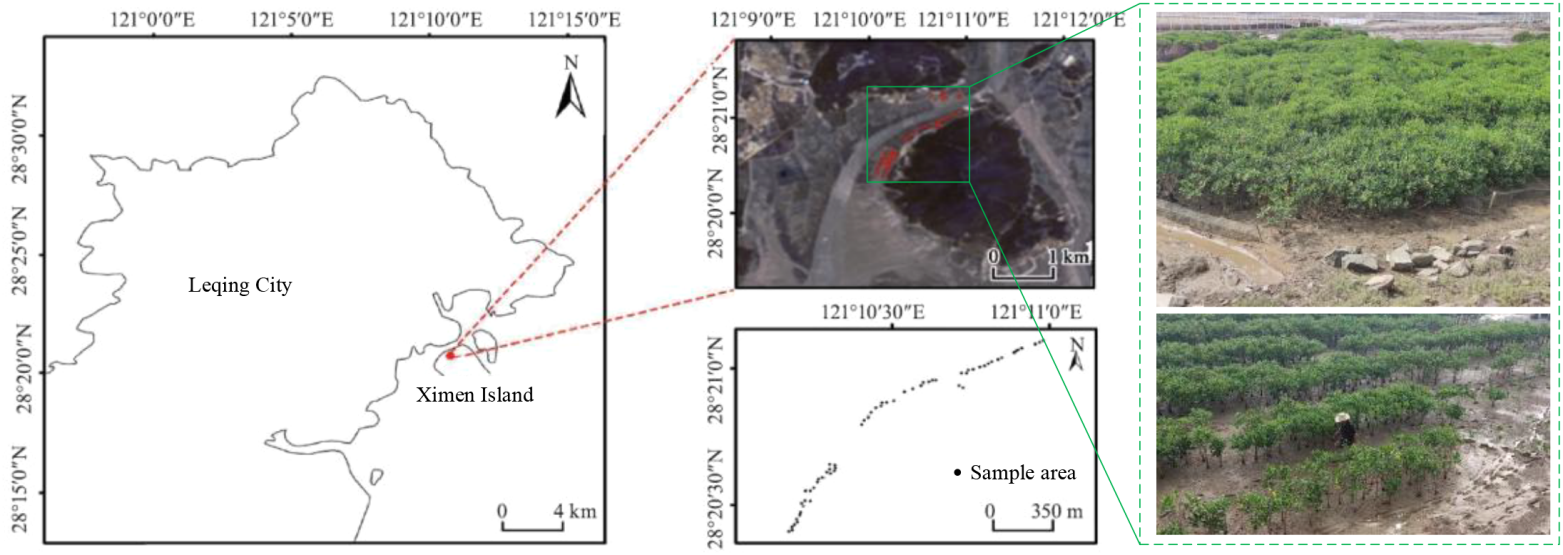
The specific location and distribution of the study area (Hao et al., 2024).

Ximen Island covers a land area of 6.98 km^2^ and a mudflat area of 15.11 km^2^. The average annual temperature is approximately 18.3°C, with an annual precipitation of 1,595.7 mm and an average annual sunshine duration of 1,714.6 hours. The mangrove wetland in this region serves as the ecological restoration project area for the coastal mangrove wetlands of Leqing City, encompassing an area of approximately 28.96 hectares for mangrove planting and 4.57 hectares for introduced mangrove maintenance (Hao et al., 2024). The plant species in this area is the *Kandelia obovata*. The location of the study area and sample distribution are shown in **Figure 4**. Landsat 8 remote sensing image data (with a spatial resolution of 30 m) from August 2022 are used in this study to map the mangrove biomass. The 21 features, including band information, vegetation indexes, texture features, and elevation features, are extracted from Landsat 8 remote sensing images. In addition, DEM data are derived from Aster GDEM with a resolution of 30 m, which is used to extract the altitude index (i.e., topographic factor). All 22 input features are presented in **Table 2**.

**Table 2:** Details about all 22 input features.

Data type	Feature description	Detailed features
Landsat 8 remote sensing data	Band	Coastal band, super blue infrared, sum green index, red band, near-infrared wave, short-wave infrared 1, short-wave infrared 2
	Vegetation indexes	Normalized difference vegetation index (NDVI), ratio vegetation index (RVI), difference vegetation index (DVI), soil-adjusted vegetation index (SAVI), enhanced vegetation index (EVI), green normalized difference vegetation index (GNDVI)
	Texture features	Variance (VAR), homogeneity (HOM), contrast (CON), heterogeneity (HET), entropy (ENT), angular second moment (ASM), correlation (COR), mean (MEA)
Digital elevation model data	Altitude index	Topographic factor (TOF)

The biomass equation (Equation 15) based on the stem diameter of the near-ground branches at the base of *Kandelia candel* is used to calculate the biomass:


(15) $$y=3.614\times D^{1.446}(R^{2}=1.801,P<0.01)$$

where *y* is the total biomass of kandelia samples and *D* is the branch trunk diameter near the ground of kandelia.

#### 3.1.2 Parameter setting

The network structure and parameters of SSDFRN are listed in **Table 3**, where *W* is the length of the shuffle window; *V*_1_, *V*_2_, and *V*_3_ are convolution kernel size of small-, medium-, and large-view branches, respectively; *S*_1_, *S*_2_, and *S*_3_ represent different sliding strides; *K* refers to the number of convolution kernels; *PW* and *PS* are the pooling window and the pooling stride, respectively; *N* is the number of neurons; and *V_de_* and *S_de_* are decoder convolution kernel size and convolution stride, respectively.

**Table 3:** Detailed structure and parameters of SSDFRN.

Module	Structure	Parameters
Input	–	–
Feature disturbing	–	*W* = 4
MVCNN	MVCCM 1	*V*_1_ = 2, *V*_2_ = 3, *V*_3_ = 4, *S*_1_ = 1, *S*_2_ = 2, *S*_3_ = 3, *K* = 16
	Pooling 1	*PW* = 2, *PS* = 2
	MVCCM 2	*V*_1_ = 2, *V*_2_ = 3, *V*_3_ = 4, *S*_1_ = 1, *S*_2_ = 2, *S*_3_ = 3, *K* = 32
	Pooling 2	*PW* = 2, *PS* = 2
Flatten	Fully connected layer	*N* = 928 − 18
Simplified decoder	Convolution 1	*K* = 16, *V_de_* = 3, *S_de_* = 1
	Pooling 1	*PW* = 2, *PS* = 2
	Convolution 2	*K* = 32, *V_de_* = 3, *S_de_* = 1
	Pooling 2	*PW* = 2, *PS* = 2
Biomass estimator	Fully connected layer	*N* = 200 − 1
Batch size = 20, learning rate = 0.0001		

SSDFRN, self-supervised disturbing feature reconstruction network; MVCCM, multi-view cascaded convolution module.

### 3.2 Experimental result

**Figure 5:**
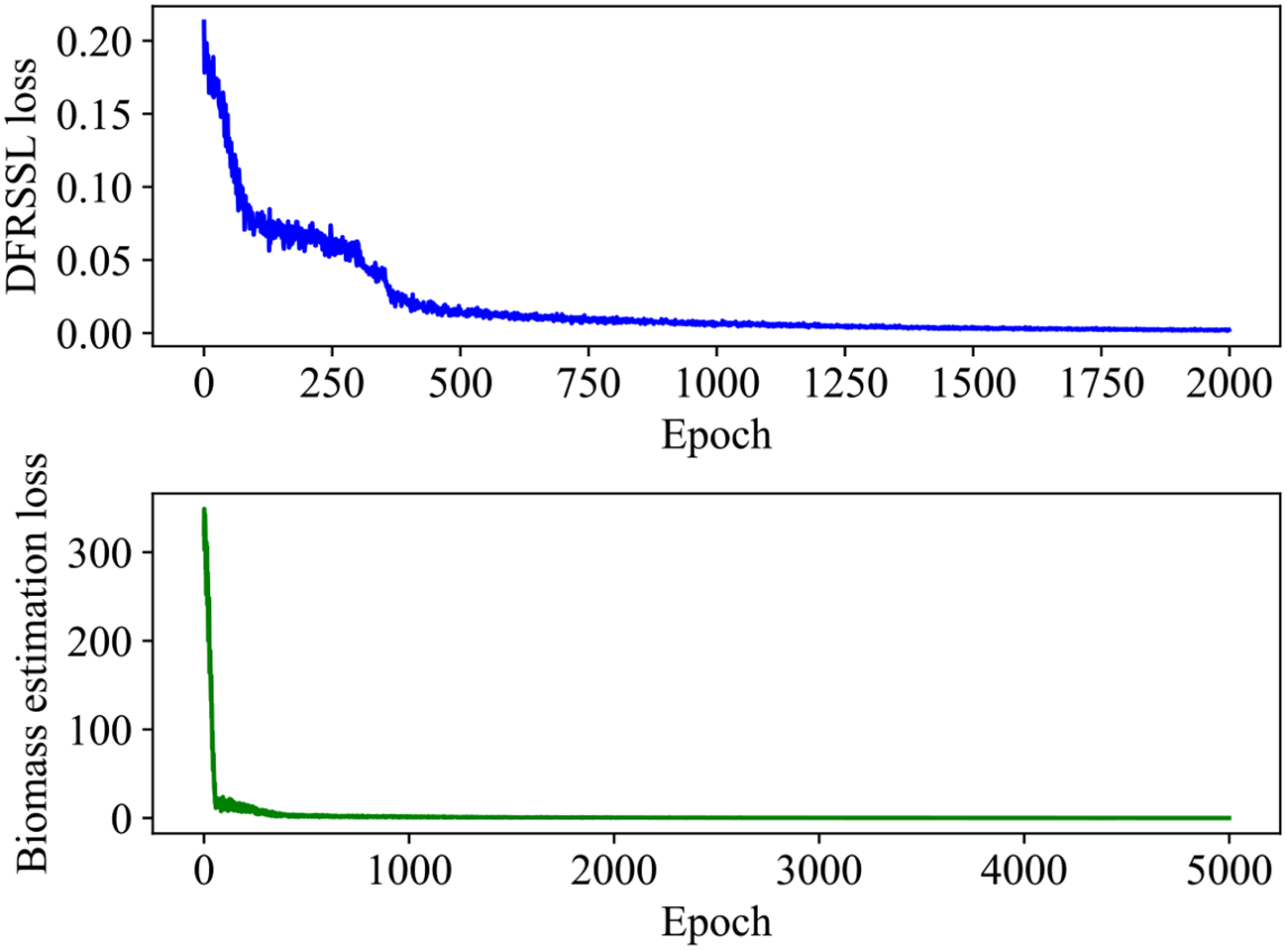
The training process of SSDFRN. SSDFRN, self-supervised disturbing feature reconstruction network.

**Figure 6:**
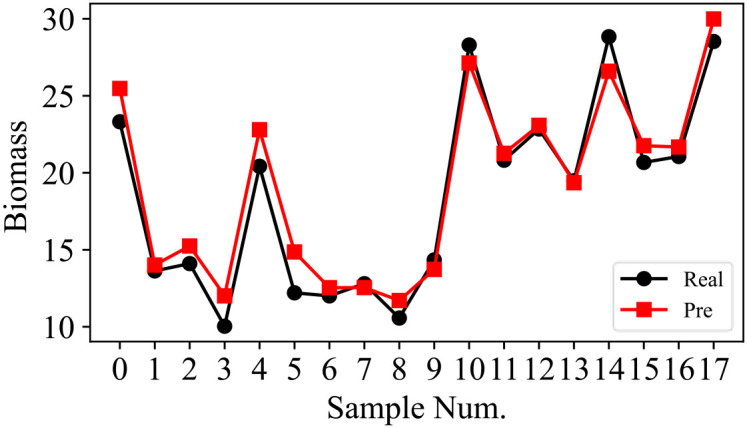
The SSDFRN-based mangrove biomass estimation results of testing samples. SSDFRN, self-supervised disturbing feature reconstruction network.

In this study, a total of 58 samples are considered in the experiment, where 70% of the samples are used for training and the remaining 30% of the samples are used for testing. Details about the dataset are presented in **Table 4**. The training process of SSDFRN is shown in **Figure 5**. It is clear that with the increase in training epochs, the DFRSSL loss and mangrove biomass estimation loss are decreasing. When the training epoch reaches 1,000, all losses are nearly zero, which means that SSDFRN exhibits excellent biomass estimation performance on the training set. The mangrove biomass estimation results on the testing set are shown in **Figure 6**. The detailed estimation results are presented in **Table 5**, where AE, MAE, and RMSE denote the absolute error, mean absolute error, and root mean square error, respectively. It is obvious that the estimated mangrove biomass values are close to the actual mangrove biomass values, which indicates the outperformance of SSDFRN on deep feature learning and mangrove biomass estimation with limited samples.

**Table 4:** Detailed information about the mangrove biomass dataset.

Samples	Sample number	Geographical position	Data shape
Total samples	58	(28° 21′ N, 121° 10′ E)	[1×22]
Training	40		
Testing	18		

**Table 5:** Detailed testing results.

Biomass	Sample Num.																	
	0	1	2	3	4	5	6	7	8	9	10	11	12	13	14	15	16	17
Actual	23.314	13.628	14.103	10.036	20.418	12.205	12.006	12.796	10.568	14.34	28.298	20.841	22.841	19.479	28.834	20.671	21.057	28.526
Estimation	25.475	14.01	15.244	12.005	22.799	14.859	12.537	12.546	11.705	13.724	27.128	21.265	23.081	19.353	26.593	21.758	21.673	29.987
AE	2.161	0.382	1.141	1.969	2.381	2.654	0.531	0.25	1.137	0.616	1.17	0.424	0.24	0.126	2.241	1.087	0.616	1.461
MAE	1.145																	
RMSE	1.396																	

AE, absolute error; MAE, mean absolute error; RMSE, root mean square error.

**Figure 7:**
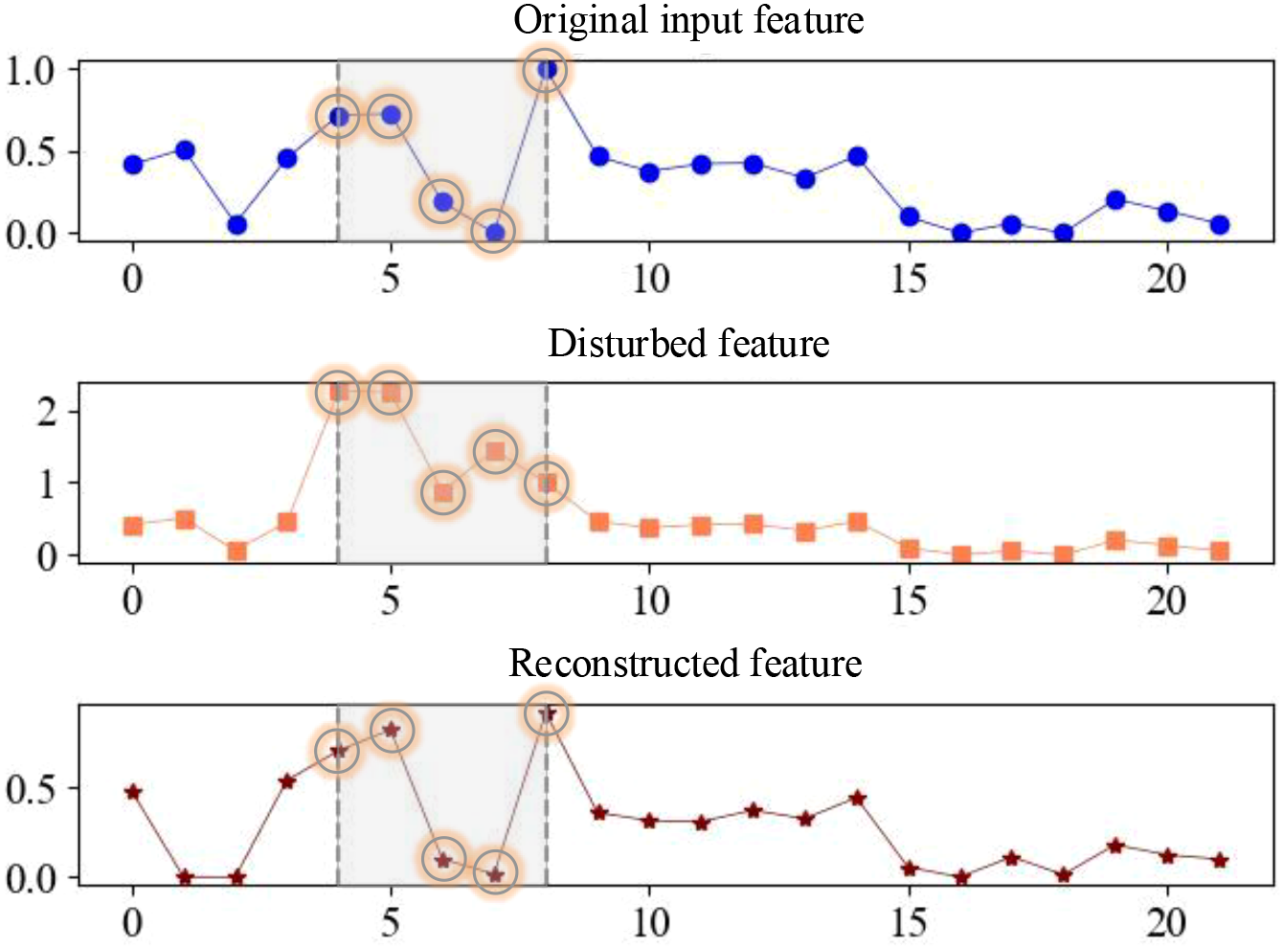
The visualization of input features, disturbed features, and reconstructed features.

In order to verify the effectiveness of DFRSSL, the original input features, disturbed features, and the corresponding reconstructed features obtained by DFRSSL are visualized in this study, as shown in **Figure 7**. It can be found that parts with original input features are shuffled and drowned by strong noise (gray parts) after the feature disturbance. After DFRSSL, noise features are greatly reconstructed by SSDFRN, which demonstrates the generalizability of SSDFRN under external interference. It indicates that SSDFRN is good at deep feature learning from limited samples based on DFRSSL.

### 3.3 Ablation study

In this section, the ablation study is implemented to verify the effectiveness of DFRSSL and MVCNN. The description of different tasks is given in **Table 6**, where “√” and “X” denote that the corresponding module is included and excluded in the task, respectively. The testing results are shown in the last two columns of **Table 6**. It is clear that the mangrove biomass estimation errors (i.e., MAE and RMSE) increase significantly when DFRSSL or MVCNN is removed from SSDFRN. It indicates that DFRSSL and MVCNN greatly enhance the feature learning and mangrove biomass estimation performance of SSDFRN.

**Table 6:** Different tasks in ablation study.

Task no.	Modules		Results	
	DFRSSL	MVCCM	MAE	RMSE
T1	✓	✓	1.145	1.396
T2	✓	X	1.367	1.660
T3	X	✓	1.250	1.626

DFRSSL, disturbing feature reconstruction-based self-supervised learning; MVCCM, multi-view cascaded convolution module; MAE, mean absolute error; RMSE, root mean square error.

### 3.4 Result comparison and discussion

**Figure 8:**
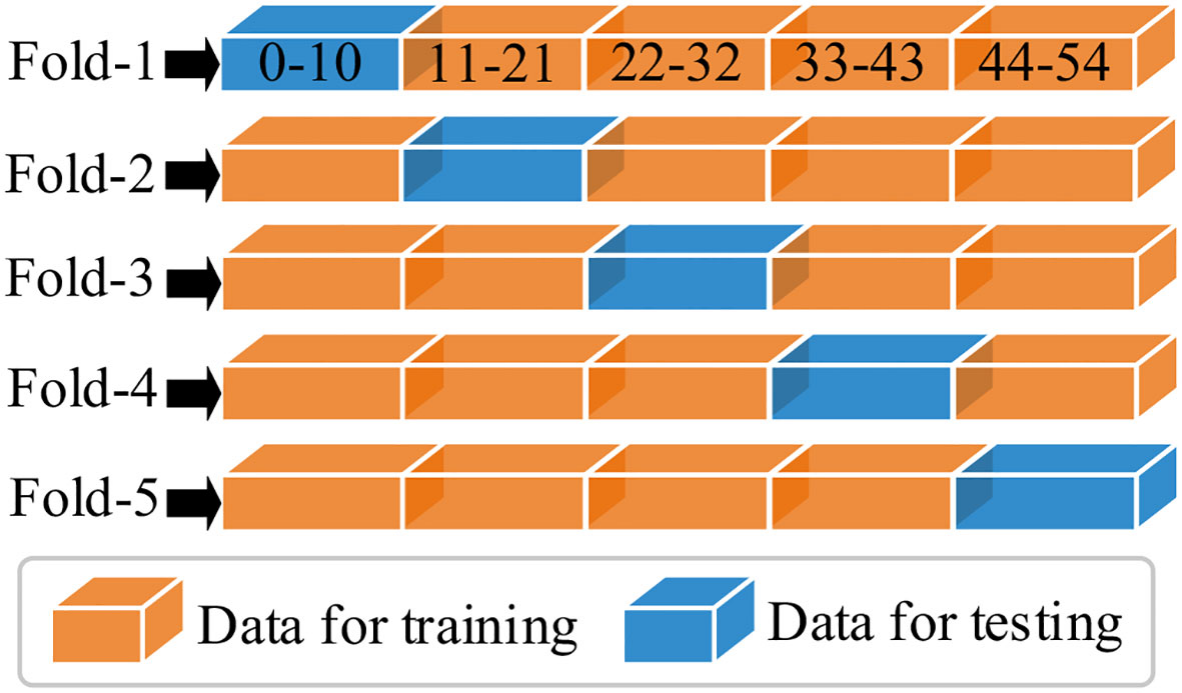
Data separation for different folds.

**Figure 9:**
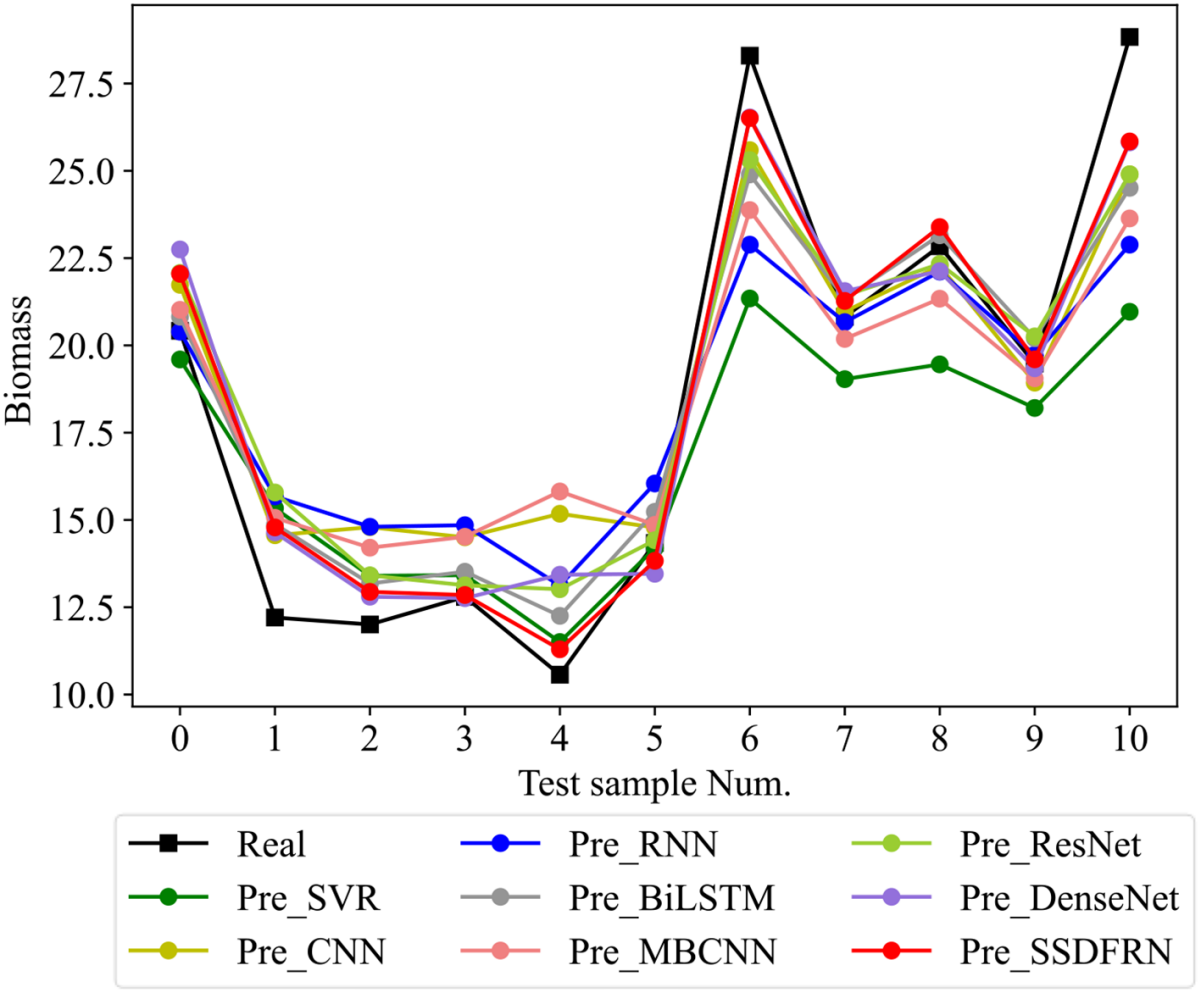
Mangrove biomass estimation results of different methods.

**Figure 10:**
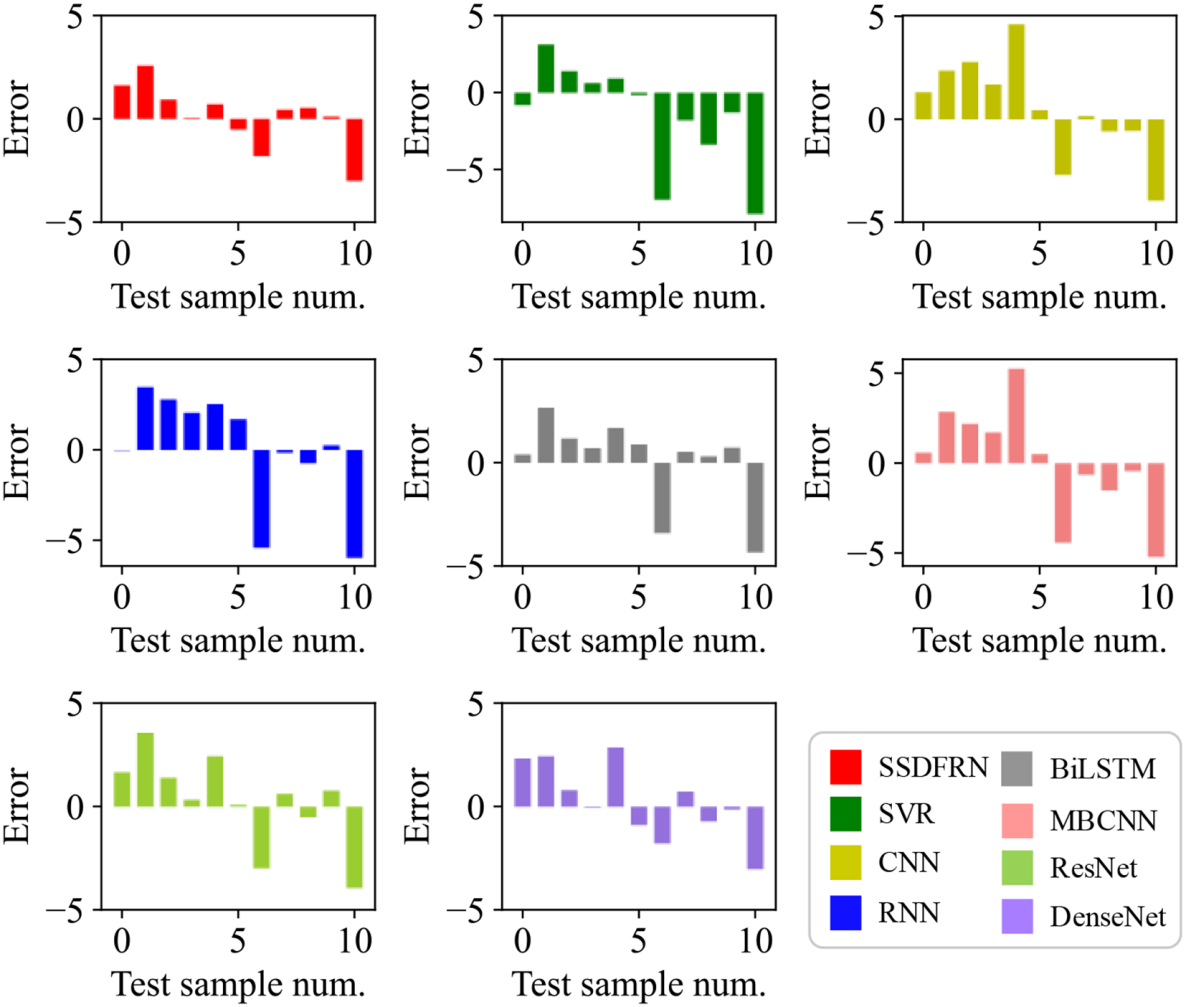
Mangrove biomass estimation errors.

In this section, the feature learning and mangrove biomass estimation performance of SSDFRN are compared with that of state-of-the-art methods [i.e., SVR (Luo et al., 2024), CNN (Nakajima et al., 2023), RNN (Song and Wang, 2023), BiLSTM (Zhang et al., 2024), ResNet (Liu et al., 2024), multi-branch convolutional neural network (MBCNN) (Zhao et al., 2021), and densely connected convolutional network (DenseNet) (Huang et al., 2017)]. As presented in **Figure 8**, fivefold cross-validation is used for data separation, where 80% of the original samples are used for training and the remaining 20% of the samples are used for testing. The computational efficiency analysis is implemented for different DNNs, and the comparison results are listed in **Table 7**. It is evident that the running time of SSDFRN is a little higher than that of other methods, and the parameter complexity and memory occupation are comparable to those of most methods, which is satisfactory for practical applications. The comparison results based on the fivefold cross-validation method are shown in **Table 8**. Taking fold-5 as an example, the mangrove biomass estimation results are given in **Figure 9**, and the estimation errors are shown in **Figure 10**. It is obvious that the mangrove biomass values estimated by SSDFRN are closer to the actual values compared with those of other methods. In particular, the mangrove biomass estimation error of SSDFRN on samples 6 and 10 is significantly smaller than that of other methods, which demonstrates the outperformance of SSDFRN on deep feature learning and mangrove biomass estimation with limited samples.

**Table 7:** Computational efficiency comparison of different DNNs.

Model	Runtime (s)	Parameter complexity (Mega Floating-Point Operations Per Second (MFLOPs))	Memory footprint (MB)	GPU utilization (%)
SSDFRN	8.71	10.54	2,242.58	31.55
CNN	6.89	6.72	2,203.80	10.50
RNN	6.62	4.09	1,403.93	14.36
LSTM	7.27	10.75	1,424.23	18.84
MBCNN	7.36	10.17	2,209.74	20.33
ResNet	7.33	7.17	2,211.09	21.87
DenseNet	7.35	10.97	2,204.02	17.00

DNNs, deep neural networks; SSDFRN, self-supervised disturbing feature reconstruction network; CNN, convolutional neural network; RNN, recurrent neural network; LSTM, long short-term memory; MBCNN, multi-branch convolutional neural network; ResNet, residual neural network; DenseNet, densely connected convolutional network.

**Table 8:** Fivefold cross-validation-based comparison results.

Method	Fold-1		Fold-2		Fold-3		Fold-4		Fold-5		Avg	
	MAE	RMSE	MAE	RMSE	MAE	RMSE	MAE	RMSE	MAE	RMSE	MAE	RMSE
SSDFRN	**1.236**	**1.535**	**1.231**	**1.632**	**1.007**	**1.201**	**1.281**	**1.487**	**1.123**	**1.467**	**1.176**	**1.464**
SVM	2.146	2.323	2.83	3.675	2.233	2.86	2.32	2.905	2.574	3.571	2.421	3.067
CNN	1.352	1.776	1.599	1.878	1.6	1.889	1.667	1.997	1.921	2.391	1.628	1.986
RNN	1.753	1.863	2.204	2.832	1.352	1.518	1.877	2.368	2.281	2.997	1.893	2.316
LSTM	1.345	1.572	1.716	2.208	1.643	1.872	1.55	1.766	1.53	2	1.557	1.884
MBCNN	1.663	1.958	1.911	2.298	1.124	1.353	1.659	1.956	2.302	2.916	1.732	2.096
ResNet	1.459	1.677	1.745	2.185	1.092	1.330	1.497	1.637	1.666	2.117	1.492	1.789
DenseNet	1.321	1.605	1.706	2.037	1.468	1.782	1.528	1.738	1.430	1.766	1.491	1.786

MAE, mean absolute error; RMSE, root mean square error; SSDFRN, self-supervised disturbing feature reconstruction network; SVM, support vector machine; RNN, recurrent neural network; LSTM, long short-term memory; MBCNN, multi-branch convolutional neural network; ResNet, residual neural network; DenseNet, densely connected convolutional network.

## 4 Conclusions

In this study, a novel DNN, i.e., SSDFRN, is developed for mangrove biomass estimation with limited data. DFRSSL is implemented by random feature shuffle and disturbing feature reconstruction, which effectively solves the key problem of data scarcity. In particular, the shuffle window is masked on partial input features randomly for generating sufficient auxiliary data and residual data. The network is pre-trained by disturbing feature reconstruction for deep feature learning using sufficient auxiliary data and residual data. In addition, a novel feature extractor, i.e., MVCNN, is constructed by stacking several MVCCMs, which effectively enhances the feature learning performance and improves the mangrove biomass estimation accuracy. The outperformance of SSDFRN is verified on the mangrove biomass dataset obtained from Ximen Island (28° 21′ N, 121° 10′ E). The testing results illustrate that SSDFRN can effectively perform deep feature learning and mangrove biomass estimation with limited data. However, the hyperparameters of SSDFRN are determined manually in this study, which is inconvenient and needs to be improved in the future. Moreover, the length of the masking window in SSDFRN is fixed, which may result in the excessive destruction of key features and may increase the difficulty of feature learning. Concurrently, the contribution of non-key features to the masking operation is inefficient, which requires further study. The future work will strive to collect more data from different study areas to underpin subsequent research and focus on improving the generalization performance of SSDFRN for different mangrove species and geographical positions simultaneously.

## Data Availability

The datasets presented in this article are not readily available because they are confidential. Requests to access the datasets should be directed to the corresponding author.
